# The mediating role of service innovation in the relationship between customer orientation and patient satisfaction

**DOI:** 10.1186/s12913-025-12794-7

**Published:** 2025-06-04

**Authors:** Joseph G. Yeboah, Kojo Tei Amponsah

**Affiliations:** Department for Entrepreneurship and Supply Chain Management, Methodist University, Accra, Ghana

**Keywords:** Patient satisfaction, Patient orientation, Service innovation, Cross-sectional survey, Service quality, Healthcare practices, Service delivery

## Abstract

**Introduction:**

Declining patient satisfaction signals a pressing challenge in healthcare service quality; however, the role of service innovation in addressing this issue remains underexplored. While patient satisfaction is the cornerstone of effective healthcare delivery, evolving patient needs and expectations demand that providers innovate to remain competitive and responsive to patient needs. Although prior research underscores the positive impact of service innovation on patient satisfaction, a critical gap persists in understanding how service innovation mediates the relationship between customer orientation and patient satisfaction. This study aims to fill this gap by examining the interplay between these factors and offering insights to enhance healthcare service strategies.

**Methods:**

A cross-sectional survey strategy was employed to gather primary data from private and public healthcare facilities in the Greater Accra and Ashanti Regions. The three largest government hospitals and the two largest private hospitals in each region were selected, due to their high referral rates. Using Snedecor and Cochran’s formula, a sample size of 386 was determined; however, this was increased to 700 to account for potential non-responses and incomplete questionnaires, yielding a final response rate of approximately 91%.

**Results:**

The results confirmed that customer orientation positively impacted service innovation (β = 0.020, *p* = 0.040), but service innovation did not significantly enhance patient satisfaction (β = 0.360, *p* = 0.560). Service innovation was not a significant mediator of the relationship between customer orientation and patient satisfaction (indirect effect β = 0.360, *p* = 0.563).

**Conclusions:**

Although customer orientation significantly enhances service innovation in healthcare, service innovation alone does not substantially affect patient satisfaction. This challenges the widely held assumption that service innovation translates to higher levels of patient satisfaction. Therefore, the pathway through which customer orientation affects patient satisfaction may be diverse and requires further research. These findings highlight the need for a holistic approach that integrates innovative services and processes with high-quality service delivery and patient engagement to meet patients’ expectations. These findings support the incorporation of patient perspectives into healthcare practices to boost service delivery and enhance patient satisfaction. Prioritising training and policies to promote a customer-centric culture to enhance customer orientation is essential for healthcare organisations.

**Supplementary Information:**

The online version contains supplementary material available at 10.1186/s12913-025-12794-7.

## Introduction

The World Health Organization indicates that quality health services must not only be universally accessible but also meet expectations and satisfy healthcare service users, particularly patients [1]. Patient satisfaction (PS), a positive evaluation of healthcare received at a healthcare facility, is an essential determinant of quality healthcare service delivery. It is a subjective evaluation of a patient’s cognitive and emotional reactions resulting from the interaction between their expectations and perceptions of the actual care received [1]. However, patient needs and expectations continue to evolve, and healthcare providers must innovate and adapt to remain competitive and meet changing demands, ultimately enhancing patient satisfaction [2, 3]. Consequently, Service Innovation (SI) has become a topic of interest in recent literature for advancing customer satisfaction [4, 5]. Notwithstanding this literature discourse, scant empirical knowledge of the implications of service innovation on customer orientation and patient satisfaction outcomes exists [6–8], particularly in sub-Saharan Africa (SSA). Concurrently, earlier studies generally focused on these constructs in isolation, leaving a dearth of empirical evidence about the interplay between Customer Orientation (CO), Service Innovation (SI), and Patient Satisfaction (PS) in healthcare.

However, recent reports have suggested that up to one-third of patients are dissatisfied with the services they receive from different healthcare facilities. Evidence from England suggests that patients have an average satisfaction score of 77.1%, indicating approximately 23% dissatisfaction [9]. In Bangladesh, evidence suggests that patients’ overall satisfaction level is 65% (51% in public and 75% in private hospitals) [10], indicating up to 35% of patient dissatisfaction. In Ghana, evidence suggests that overall patient satisfaction is approximately 68.97% [11], indicating that more than 30% of patients are dissatisfied. Given that healthcare concerns human well-being and life, it must be mentioned that this statistic reflects the myriad people with unmet needs, which may be life-threatening.

Dissatisfaction with healthcare services is often a consequence of several factors, including poor patient handling, interaction, and a poor environment, which may lead to poor treatment outcomes [10, 11]. These outcomes may include delayed or inadequate medical attention, exacerbating health conditions and complications. Additionally, poor communication or interaction between patients and healthcare providers, often from dissatisfaction, can contribute to misunderstandings regarding diagnoses, treatment options, and follow-up care, leading to suboptimal health outcomes. Addressing these issues requires healthcare organisations to pay critical attention to customers’ perspectives, necessitating the need to emphasise customer orientation in healthcare service delivery.

Customer orientation is considered an essential and integral element of modern organisations, particularly in healthcare service delivery. Healthcare providers, like any enterprise, rely on direct interactions with their patients, who ultimately drive the development and refinement of the services they offer [12, 13]. The needs and preferences of patients shape the primary direction of growth for healthcare organisations because patients’ well-being is the primary mandate [14, 15]. Customer orientation projects patients’ unique requirements and expectations, which are factored into healthcare service delivery to enhance patient satisfaction. Therefore, understanding and prioritising patient needs is crucial for healthcare providers to improve patient satisfaction and outcomes. This customer-centric approach aligns with the broader principles outlined by the World Health Organisation [16]. Hence, customer orientation influences healthcare strategies, such as implementing innovative techniques to enhance service delivery and boost patient satisfaction.

Therefore, service innovation can be a direct consequence of customer orientation. Service innovation, defined as the implementation of new or significantly improved service processes, techniques, or practices, plays a crucial role in customer satisfaction [17, 18]. The nature of innovative healthcare services reflects patient needs, which are best understood when the healthcare system operates from a customer-oriented perspective. For instance, innovations such as telemedicine enhance service delivery by addressing patient needs for increased convenience, personalised treatment plans, and streamlined patient communication systems. Some studies have further linked service innovation to customer satisfaction, emphasising that improved customer satisfaction implies practical and adequate service innovation [19]. Therefore, service innovation is a significant factor in customer orientation and patient satisfaction.

Therefore, patient satisfaction is a key determinant of healthcare service quality, influenced by customer orientation and service innovation. However, limited research has examined their combined effects on patient satisfaction in healthcare. While studies have highlighted the impact of SI on customer satisfaction [13, 20], most existing research is based on non-healthcare industries such as retail and automotive [21]. The SI has been found to enhance customer satisfaction directly and mediate the relationship between service delivery, satisfaction, and loyalty [22, 23]; however, similar studies in healthcare remain limited.

Despite the acknowledged relevance of SI in improving healthcare quality, little empirical attention has been paid to this field. A few existing studies have analysed the effect of SI on patient satisfaction and loyalty, with some showing its positive impact during the COVID- 19 pandemic [13, 24]. However, none of these studies have considered how SI mediates the relationship between customer orientation and patient satisfaction, particularly in Ghana and Sub-Saharan Africa. Since healthcare service quality remains a significant concern in the region, addressing this gap is crucial for achieving the x (SDGs).

This study sought to address these gaps by first understanding (1) how CO influences SI in healthcare services (2). How does SI impact PS in healthcare services (3)? What is the mediating effect of SI on the relationship between CO and PS in healthcare service delivery?

Understanding the relationship between customer orientation, service innovation, and patient satisfaction is important for policymakers, healthcare administrators and service providers. By cultivating a robust customer-centric culture and utilising modern healthcare solutions, hospitals and clinics can elevate patient satisfaction and boost overall healthcare service delivery. Policymakers can use these findings to design regulatory frameworks promoting service innovation while safeguarding patient-centred care. Furthermore, healthcare managers can execute strategic efforts that synchronise innovation with patient expectations, enhancing healthcare outcomes and improving system efficiency. This study offers significant evidence to inform decision-making and policy development for sustainable healthcare enhancements, addressing the ongoing difficulties in service quality throughout sub-Saharan Africa.

## Literature review

### Customer orientation and service innovation

The literature recognises that two key elements (CO and SI) have the potential to enhance service quality in various sectors, including health care. CO refers to an organisation’s capacity to recognise and satisfy consumer demands, strengthening long-standing connections with customised service offerings [13, 24]. In contrast, SI entails implementing new ideas, tools, or techniques to improve results or service delivery [25]. Although studies have shown a clear correlation between CO and organizational performance, including customer satisfaction, and the relevance of SI in improving service efficiency and customer care [7, 26], these dimensions remain underexplored in healthcare service delivery. Most research has focused on examining these variables individually, ignoring how CO affects SI acceptance and success in healthcare settings. This discrepancy calls for more empirical research, especially in the healthcare sector, where knowledge of the nature of CO’s influence on SI would provide insightful information for maximising patient treatment and service innovation.

It has been argued that organisations that prioritise CO are more likely to engage in SI because they seek to meet and exceed customer expectations through novel and improved service offerings [27, 28]. Despite these findings, some studies indicate that the relationship between customer orientation and service innovation may not be universally applicable across all sectors. For instance, in certain contexts, the direct impact of customer focus on service innovation remains inconclusive, suggesting that other strategic orientations may play a more significant role [29]. Contrary to this view, some empirical studies have found that organisations with a strong CO culture are better at gathering and utilising customer feedback, which fuels the development of innovative services [30, 31]. CO leads to a proactive stance in identifying unmet customer needs and opportunities for service improvement, thus fostering a conducive environment for SI [32]. Furthermore [33], argued that CO provides the critical market intelligence necessary for effective innovation, whereas [34] demonstrated that CO enhances organizational learning and adaptability, which are crucial for successful service innovation. Therefore, it is hypothesised that:

- (H_1_): Customer Orientation (CO) has a significant positive relationship with Service Innovation (SI).

### Service innovation and patient satisfaction

SI is increasingly recognised as a crucial factor in enhancing PS in healthcare settings [17]. Studies have shown that by meeting changing requirements and expectations, service innovation can enhance patient experience, simplify care delivery, and raise satisfaction [7, 35]. The perceived quality, responsiveness, and dependability of services shape patient satisfaction, which is a crucial healthcare outcome [36]. Although some studies have shown a correlation between general healthcare quality and innovation [32, 33], others argue that there are no particular processes by which service innovation directly affects patient satisfaction [37]. Existing research hardly investigates how specific innovative service ideas translate into higher degrees of satisfaction, particularly in patient-centred environments, where emotional and cognitive reactions to treatment are critical. Assessing the nature of the influence between these variables can help clarify the conflicting viewpoints in the literature on how service innovation affects patient satisfaction and guides evidence-based changes in patient care.

It is, however, important to reinforce that previous empirical evidence suggests that SI contributes positively to customer satisfaction by addressing customer needs more effectively and efficiently [7, 38, 39]. For instance, research has shown that innovative service practices, such as personalised care plans and advanced medical technologies, lead to higher patient satisfaction by improving the quality of care and reducing wait times [40]. Furthermore, others have highlighted that SI fosters a more patient-centred approach, thereby increasing patients’ perceived value of the services received [41]. The SI enhances patient trust and loyalty, which are critical components of overall satisfaction [42]. Therefore, this study hypothesises that:

- H_2_: SI has a significant positive relationship with PS, as it directly improves service quality and patient outcomes through innovative approaches.

### Customer orientation and patient satisfaction, and the mediating role of service innovation

The healthcare industry has increasingly recognised the importance of CO as a critical factor in enhancing PS. PS serves as a key indicator of healthcare quality, which gauges how well services either match or surpass patient expectations, thereby affecting their confidence and allegiance to healthcare providers [7]. The literature suggests that SI and PS have a close relationship, as innovations typically simplify procedures, reduce waiting times, and provide tailored treatment, all of which are fundamental to patient satisfaction [26, 35]. Another driver of satisfaction is CO, which emphasises knowledge and meeting patient demands, thereby promoting a service culture that prioritises patient well-being [36]. Recently, innovations in service delivery have become catalysts for meeting customer expectations. Consequently, existing studies have suggested that SI could potentially play a significant role in the relationship between CO and PS [6, 25]. Amidst these inferences, there is a paucity of empirical evidence showing the effect SI could have on the link between CO and PS, especially in healthcare.

Furthermore, some studies have demonstrated that a strong CO is positively correlated with higher levels of patient satisfaction [12]. However, the pathway through which CO is translated into PS often involves intermediary processes, among which, SI plays a pivotal role. Service innovation introduces new or significantly improved services, processes, or delivery methods that can enhance the overall patient experience by making healthcare services more efficient, personalised, and responsive to patient needs [17, 39, 43]. Other studies support the view that SI acts as a mediator that can amplify the positive effects of CO [20, 44]. CO affects PS by ensuring that the services provided are not only aligned with customer expectations but also exceed them through innovative approaches. Therefore, it is hypothesized that.

- (H_3_): Service Innovation (SI) mediates the relationship between Customer Orientation (CO) and Patient Satisfaction (PS).

**Fig. 1:**
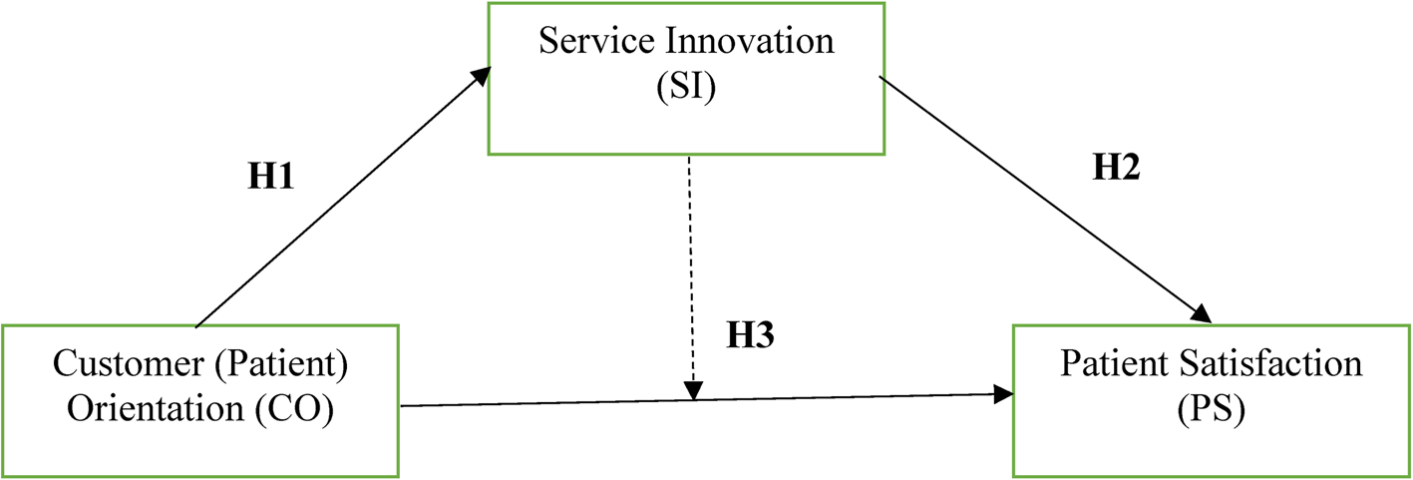
Conceptual Framework, Authors’ Construct. Bold arrows signify direct relationships, while dashed arrow signifies a mediating interaction

Figure 1 illustrates the conceptual framework of this study.

## Methods

### Study design and setting

A cross-sectional survey strategy was employed to gather primary data from patients visiting health care facilities in Ghana. This study focused on healthcare delivery facilities in the Greater Accra and Ashanti Regions, encompassing private and public hospitals. Ten hospital facilities were sampled from over 500 health facilities in the two regions. Specifically, the three largest government hospitals and the two largest private hospitals from each region were selected, as these top hospitals received the most referral cases and conditions from across the country.

### Study participants

The target population for this study comprised patients who visited health facilities at least once because of poor health, regardless of the time of visit. The sample size was calculated using Snedecor and Cochran’s formula [45]:

$$\:{\text{n}}_{\text{o}}={\text{z}}^{2}\,\text{p}\text{q}/{\text{e}}^{2},$$

where n_o_ represents sample size,

z is the confidence level,

p is the population variability,

where q is given as 1 − p, and e is the level of precision.

Using a 95% confidence level (z = 1.965), a variability of 0.5, and 5% precision, the formula yielded a sample size of 386, which is representative of large populations. However, considering that patient satisfaction surveys typically have poor response rates (50–55%) [46], the sample size was increased to 700 to account for potential non-responses and incomplete questionnaires. Of the 700 questionnaires distributed, 635 valid responses were received, resulting in a response rate of approximately 91%.

### Data collection tool and procedure

Data were gathered from participants using a self-administered structured questionnaire designed for this study based on the literature. A primary draft of the questionnaire was prepared in English, comprising two parts: Part I and II (see Supplementary file).

(i) Part I: Background information on the participants was collected through a structured questionnaire that included demographic and personal information. This section comprised seven items: gender (male or female), age group (ranging from 18 to 20 to 61 and above), education level (from illiterate to master’s degree and others), marital status (married, single, or divorced), occupation, name of the hospital visited, and the number of times the participant had visited the facility.
(ii) Part II: This section is divided into three main sections: Section A: Customer Orientation, Section B: Section C: Patient Satisfaction. Each section was designed to capture specific dimensions of patients’ experiences and perceptions of healthcare services. The questionnaire utilised a 5-point Likert scale for responses, ranging from “Strongly Disagree” to “Strongly Agree”, to quantify the participants’ agreement with various statements. This scale allows for the measurement of attitudes and perceptions in a standardised manner, facilitating data analysis.
  For Section A, which focused on Customer Orientation, the researchers constructed the scale based on the literature, specifically [47–49] as shown in Table 1. The scale comprised 10 questions to assess the extent to which patients perceived the hospital’s services as personalised, satisfactory, and responsive to their needs. The items included statements about the range of services, efforts to satisfy patients, and the hospital’s proactive approach to understanding patient needs. This section demonstrated strong internal consistency, with a Cronbach’s alpha value of 0.884, indicating the high reliability of the scale used to measure customer orientation.

**Table 1:** Development of scale for customer orientation based on literature

Critical Dimension	Item Wording	Literature Evidence
CO1	I am excited about the range of services that the hospital provides	Anderson (2006) [50]
CO2	The hospital provides personalised services that meet my health preferences	Anderson and Sullivan (1993) [51]
CO3	Customer service is provided excellently by the hospital	Anderson (2006) [50]
CO4	The hospital works hard to satisfy patients;	Narver and Slater (1990) [52]
CO5	No matter how employees feel, they always do the best they can for every patient they serve	Narver and Slater (1990) [52]
CO6	The hospital often makes an extra effort to help patients even if it is not expected of them	Narver and Slater (1990) [52]
CO7	The hospital periodically finds out patient’s needs in order to serve them better	Anderson (2006) [50]
CO8	I am well-informed about the services being provided	Narver and Slater (1990) [52]
CO9	I have high confidence in the services that the hospital provides.	Appiah-Adu (1998) [48]
CO10	The range of health services that the hospital provides is adequate.	Narver and Slater (1990) [52]
  Section B evaluated Service Innovation through ten items designed to understand patients’ perceptions of the hospital’s use of modern technology and innovative practices in service delivery. As shown in Table 2, the scale for service innovation was based on several studies [53–57]. The statements covered areas such as the use of technology in treatment routines, administrative procedures, and overall satisfaction with innovative practices. Internal consistency was measured using a Cronbach’s alpha value of 0.785, reflecting an acceptable level of reliability.

**Table 2:** Development of a scale for service innovation based on literature

Critical Dimension	Item Wording	Literature Evidence
Modern Technology	The hospital provides modern treatment routines for patients	Venkatesh et al., (2003) [47]
New Delivery System	The hospital has a technology that simplifies patients’ administration procedures	Brown & Venkatesh (2005) [58]
Modern Technology	The hospital has a modern technology that helps in diagnosing and treating patients	Venkatesh et al., (2003) [47]
New Delivery System	Hospital staff ensures that the right tools and equipment are used in the provision of service.	Bharadwaj et al., (1993) [59]
New Service Concept	The use of technology gives a priority in the provision of healthcare services at the hospital.	Caruana (2002) [60]
New Culture	I have great confidence in the systems used by the hospital in the provision of services.	Caruana (2002) [60]
New Customer Interaction	Services delivery at the hospital is fast and quick.	Claycomb & Martin (2002) [61]
New Culture	Poor systems are avoided by the hospital to ensure patient satisfaction.	Venkatesh et al., (2003) [47]
New Culture	The hospital uses innovation in the provision of services	Venkatesh et al., (2003) [47]
New Customer Interaction	I am satisfied with the innovative ways the hospital adopts in the provision of services.	Appiah-Adu (1998) [48]
  Finally, Section C focused on Patient Satisfaction and contained five items that assessed overall satisfaction with various aspects of the hospital’s services, including physician availability, administrative procedures, and treatment quality. Table 3 depicts the development of the measurement constructs based on several literature [48, 49, 53, 54]. This scale exhibited relatively high internal consistency, with a Cronbach’s alpha value of 0.921, indicating a great degree of reliability.

**Table 3:** Development of a scale for patient satisfaction based on literature

Critical Dimension	Item Wording	Literature Evidence
Patient Satisfaction	Overall, I was satisfied with the physician’s level of availability in attending to me	DiTomasso and Willard (1991) [49]
Patient Satisfaction	Overall, I was satisfied with the administrative procedures in the hospital	Chahal and Mehta (2013) [53]
Patient Satisfaction	Overall, I was satisfied with the treatment by the medical staff.	Chahal and Mehta (2013) [53]
Patient Satisfaction	I was satisfied with the quality of services that the hospital provides.	Appiah-Adu, (1998) [48]
Patient Satisfaction	I received the kind of services that I needed and preferred from the hospital.	Henkel (2006) [54]

### Statistical analysis

STATA version 16 was used for the statistical analyses. All paper-based data were cleaned, coded, and entered into STATA software (STATA/MP). This study used descriptive and inferential statistical analyses. The descriptive phase explained and analysed the patients’ demographic background. In contrast, the inferential phase tested a formulated model hypothesis using the SEM-PLS method.

### Ethical consideration

Ethical considerations for this study included obtaining informed consent from all participants, ensuring the confidentiality and anonymity of patient data, and adhering to the ethical guidelines set forth by the Institutional Review Board (IRB) of Open University Malaysia (OUM)/Accra Institute of Technology (AIT). The study therefore complied with the ethical principles required in research with human participants, as enshrined in the Declaration of Helsinki. The study ensured that no harm came to the participants, and the data were handled responsibly and securely to protect patient privacy and integrity throughout the research process.

## Results

### Demographic profile of participants

The characteristics of the patients included in this study are shown in Table 4. The study included 635 patients from public (*n* = 408) and private (*n* = 277) hospitals, with 56.4% male and 43.6% female. The age distribution showed that 36.1% were 41–50 years old and 25.2% were 31–40. Regarding marital status, 48% were married, 41.7% were single, and 10.2% were divorced. Regarding visitation, 40.9% were first-time visitors, 18.2% had 2–4 visits, and 40.9% had more than four visits. Education levels ranged from illiteracy (8.2%) to first-degree and above (18.7%).

**Table 4:** Demographics of hospital patients

	Information	Hospitals		Public Hospital		Private Hospital	
		Total Sample = 635		Sample = 408		Sample = 277	
		N	%	N	%	N	%
Gender	Male	358	56.4	219	53.7	139	61.2
	Female	277	43.6	189	46.3	88	38.8
Age	Less than 20	32	5.0	21	5.1	11	4.8
	21–30	65	10.2	42	10.3	23	10.1
	31–40	160	25.2	101	24.8	59	26.0
	41–50	229	36.1	147	36	82	36.1
	51 and above	149	23.5	97	23.8	52	22.9
Marital Status	Married	305	48.0	173	42.4	132	58.1
	Single	265	41.7	191	46.8	74	32.6
	Divorced	65	10.2	44	10.8	21	9.3
Number of Visitation	1st Visit	260	40.9	173	42.4	87	38.3
	2nd − 4th Visit	115	18.2	105	25.7	10	4.4
	Above 4th Visit	260	40.9	130	31.9	130	57.3
Education	Illiterate	52	8.2	34	8.3	18	7.9
	JHS Graduate	63	9.9	41	10	22	9.7
	SHS Grad	196	30.9	125	30.6	71	31.3
	Diploma Graduate	205	32.3	130	31.9	75	33.0
	1st Degree and above	119	18.7	78	19.1	41	18.1

Source: Field Study (2023)

### Confirmatory factor analysis (CFA)

CFA was used to confirm the appropriateness of the constructs used in this study and to ascertain whether all measures were true indicators of latent variables (CO, SI, and PS).

The model fit criteria commonly used for absolute fit are chi-square (χ²), GFI, AGFI, RMR, and RMSEA. These criteria are based on the differences between the observed and model-implied correlation or covariance matrices [55]. A comparative fit deals with whether the considered model is better than a competing model in accounting for observed data. A comparative fit assessment is based on the examination of a baseline model compared to theoretically derived models [56].

Some criteria in this category include NFI, CFI, and RNI. The following fit indices were used to evaluate how well the measurement model fit the data collected, with each having conventionally acceptable values: RMSEA ≤ 0.08, GFI ≥ 0.90, NFI ≥ 0.90, and CFI ≥ 0.90 [57]. The sufficiency of the theorised model’s creation of a covariance matrix is evaluated by the χ2 goodness-of-fit value, which also estimates the coefficients compared with the observed covariance matrix. However, because the value of χ2 is affected by the sample size, a large number of participants can inflate the χ2 when assessing the model fit [62]. In one of the more comprehensive and widely cited evaluations of cut-off criteria, the findings of the simulation studies suggested the following guidelines for acceptable model fit: (a) SRMR values close to 0.08 or below; (b) RMSEA values close to 0.06 or below [62]. Nonetheless, some literature also indicates that the cutoff point for RMSEA values should be less than 0.08, and (c) CFI and TLI values should be close to 0.95 or greater [63].

The CFA outputs show that the values or estimates obtained for the various measures (SRMR, TLI, CFI, and RMSEA) in the confirmatory factor analysis largely met the threshold criteria. Tables 5, 6 and 7 present the confirmatory factor analysis results for customer orientation, service orientation, and patient satisfaction.

**Table 5:** Fitness threshold (customer orientation)

Measures	Criteria	Study Outputs (Estimates)
SRMR	Close to 0.08 or below	0.022
TLI	Close to 0.95 or greater	0.982
CFI	Close to 0.95 or greater	0.988
RMSEA	Close to 0.08 or below	0.072

**Table 6:** Fitness threshold (service innovation)

Measures	Criteria	Study Outputs (Estimates)
SRMR	Close to 0.08 or below	0.010
TLI	Close to 0.95 or greater	0.984
CFI	Close to 0.95 or greater	0.991
RMSEA	Close to 0.08 or below	0.072

**Table 7:** Fitness threshold (patient satisfaction)

Measures	Criteria	Study Outputs (Estimates)
SRMR	Close to 0.08 or below	0.007
TLI	Close to 0.95 or greater	1.000
CFI	Close to 0.95 or greater	1.000
RMSEA	Close to 0.08 or below	0.000

### Validity and reliability test

The reliability measures were above acceptable satisfactory levels (Cronbach’s alpha > 0.70, Average Variance Extracted > 0.50, and composite reliability > 0.70), as recommended by scholars [64, 65]. The factor loadings also indicated good convergent validity. Thus, the measurement model presented in Table 8 is regarded as one that adequately fits the data for this study.

**Table 8:** Validity and reliability test for CFA model

**Construct**	**Construct validity**		**Construct reliability**	
	**Factor loadings**	**AVE**	**Composite reliability**	**Cronbach alpha**
Customer orientation		0.748	0.967	0.967
CO1	0.882			
CO2	0.681			
CO3	0.861			
CO4	0.885			
CO5	0.907			
CO6	0.675			
CO7	0.917			
CO8	0.936			
CO9	0.911			
CO10	0.940			
Service innovation		0.831	0.98	0.979
SI1	0.904			
SI2	0.921			
SI3	0.914			
SI4	0.904			
SI5	0.893			
SI6	0.923			
SI7	0.93			
SI8	0.923			
SI9	0.891			
SI10	0.914			
Patient satisfaction		0.709	0.732	0.924
PS1	0.785			
PS2	0.891			
PS3	0.909			
PS4	0.833			
PS5	0.785			

### Discriminant validity

Discriminant validity ensures that a constructed measure is empirically unique and represents the phenomenon of interest that other measures in a structural equation model do not capture [66]. This study adopted Fornell and Larcker’s criterion to check validity [65]. Therefore, each construct’s AVE must be compared with the squared correlations between each pair of variables. The square root of AVEs that is greater than any squared correlation, suggesting that discriminant validity has been achieved [65].

Likewise, this method compares the square root of the average variance extracted (AVE) with the correlation of the latent constructs [55]. A latent construct should explain the variance of its indicator better than that of other latent constructs. Therefore, the square root of each construct’s AVE should be greater than the correlations with other latent constructs [55].

From Table 9, it is evident that all the squared roots of the AVEs of the constructs are greater than the correlation of the constructs. This means that discriminant validity was established, implying that the latent variables were valid and unique.

**Table 9:** Fornell and Larcker-Discriminant validity

Variables	CO	SI	PS
CO	0.865		
SI	− 0.005	0.912	
PS	0.053	0.481	0.842

### Assessment of hypothesis

The structural model used in this study was intended to test hypothetical propositions based on the conceptual framework. The conceptual model is evaluated in terms of measures of fit, statistical significance of coefficients, and interpretation, following the summarised results of the hypothesis test in Table 10. Subsequently, the mediating role of service innovation was tested by examining its direct and indirect effects on customer orientation and patient satisfaction.

**Table 10:** Assessment of structural model (Hypothesis testing: H_1_, H_2_)

Direct Path	Coeff	Z	*p* > [z]
H_1_, CO -> SI	0.020	0.58	0.040
H_2_, SI -> PS	0.360	12.40	0.563

chi^2^ (623) = 3247.87, Prob > chi^2^ = 0.0000, R^2^ = 0.98, *n* = 635

#### H_1_: customer orientation (CO) has a significantly positive relationship with service innovation (SI)

Hypothesis H_1_ is represented by the coefficient of the path CO - SI. The findings revealed that customer orientation positively relates to service innovation. As shown in Table 10, the results indicate that the estimated customer orientation-to-service innovation is statistically significant at a p > [Z] value of 0.040. This finding confirms that a 1% variation or change in customer orientation is likely to improve service innovation by 2.0%. Furthermore, a *p*-value of 0.00 < 0.05 assumes a positive significant relationship between Customer Orientation and Service Innovation.

#### H_2_: service innovation (SI) has a positive and significant relationship with patient satisfaction (PS)

The following hypothesis was formulated to test the claim that service innovation (SI) has a positive effect on perceived patient satisfaction (PS) in Ghanaian hospitals. Hypothesis H2 is represented by the coefficient of the path SI → PS. The standardised path coefficient of service innovation was 0.360 with a Z value of 12.40, at the p > [Z] of 0.563, as shown in Table 10. The results show that service innovation is positively related to patient satisfaction. However, the *p*-value (0.563 > 0.05) indicates a positive but weak relationship between service innovation and patient satisfaction.

#### H_3_: service innovation (SI) mediates the relationship between customer orientation (CO) and patient satisfaction (PS)

Table 11 presents the results for H_3,_ represented in the model by the coefficient of the path CO → SI → PS. Path CO - SI was significant (Coeff = 0.020 and *p* = 0.040), but the direct effect of service innovation on patent satisfaction, which is the standardised path coefficient, was not significant (SI → PS) at 0.360 and *p* = 0.563. Only the direct path (CO -> SI) was significant (*p*-value for direct 0.040 < 0.05, indirect 0.563 > 0.05), which implies that there is no mediation (Table 11).

**Table 11:** Test of mediational effect

Structural Path	Direct Effect	Direct Effect	Mediation Effect
H_3_, CO -> SI -> PS	0.020 (0.040)	0.360 (0.563)	No mediation

## Discussion

This study investigated the mediating role of service innovation in the relationship between customer orientation and patient satisfaction. The findings indicate that while customer orientation positively affects service innovation (H_1_), service innovation does not significantly affect patient satisfaction (H_2_). Thus, the mediating effect of service innovation on the customer orientation-patient satisfaction relationship was not supported (H_3_).

This study underscores that customer orientation significantly boosts service innovation, corroborating the findings of [67] and the principles of customer-centric approaches described by [19]. This emphasises the importance of addressing customer needs and expectations, which are vital for developing innovative treatment methods and administrative processes in the future. Although prior studies [7, 68] have demonstrated that service innovations, such as telemedicine and personalised care plans, can enhance patient satisfaction, the findings of this study indicate that such innovations alone do not significantly increase patient satisfaction. This deviation suggests that service innovation, despite its operational benefits, does not enhance patient satisfaction universally. A likely explanation lies in the unique complexity of the healthcare sector: unlike commercial industries, where satisfaction often correlates with novel services or technologies [20, 44], healthcare satisfaction is deeply tied to relational and emotional factors such as trust in providers, perceived empathy, and personalised care [69–72].

Similarly, in contrast to the findings [73], which highlighted the benefits of service innovation in increasing patient satisfaction, this study revealed that broader service delivery and patient engagement were pivotal in increasing patient satisfaction. As [74] noted, factors such as respect for patient dignity, perceived quality of basic services, and the overall hospital environment play a significant role in patient satisfaction. Innovations that prioritise efficiency and standardisation (without addressing human-centred elements) may fail to resonate with patients. Simply integrating service innovations, such as streamlined administrative processes and advanced medical equipment, may not lead to higher patient satisfaction if these do not meet patient expectations and if the quality of interpersonal interactions is not maintained. Again, this disparity can be due to varying patient expectations, especially along levels of development. In emerging countries with fundamental healthcare system issues, patients may prioritise essential factors such as accessibility, price, waiting periods, and direct engagement with physicians over technological or administrative advancements [75–78].

Hence, service innovations that do not address these fundamental concerns will have minimal effects on patient satisfaction. Service innovation initiatives that fail to integrate well with patient-centred care may be regarded as impersonal, resulting in disconnection between healthcare providers and patients. This indicates that for service innovation to effectively enhance patient satisfaction, it must be part of a comprehensive approach that encompasses high-quality service delivery, active patient engagement, and consistent communication, reflecting the complex nature of patient satisfaction in healthcare. This underscores the need for healthcare providers to balance technological advancements with strategies that preserve interpersonal connections and address diverse patient expectations.

This study contributes to the understanding of how customer orientation fosters service innovation in healthcare. This finding suggests that merely implementing service innovation and processes may not be sufficient to enhance patient satisfaction without addressing patient engagement and service quality. This finding advances the current view by highlighting the need for a holistic approach that integrates customer orientation, quality service delivery, and patient engagement to improve the patient satisfaction.

This study had some limitations. First, the study utilised self-reported questionnaires, which may have introduced bias. This cross-sectional design also limits the ability to draw causal inferences. Future studies should employ longitudinal designs and consider moderating variables such as patient engagement and quality of service delivery. Additionally, specific healthcare facilities, such as the differences between public and private hospitals, could influence the outcomes and should be examined further.

## Conclusion

While it is widely acknowledged that service innovation results in enhanced customer satisfaction, this study presents a contrary view, indicating the need to consider other factors that may be at play in ensuring patient satisfaction. Nevertheless, the impact of customer orientation on service innovation cements the crucial role of patient-centredness in healthcare service delivery. This highlights the need for policies and training to establish a customer-centric culture in healthcare settings. The pathway from customer orientation to patient satisfaction requires further investigation, given that service innovation may not be an adequate link. Future studies should explore other relevant mediating and moderating factors, notably the relationship between customer orientation and specific types of service innovation. The influence of service innovation on patient engagement and service delivery outcomes should be explored.

### Implications

The study’s findings indicate that service innovation alone is inadequate for improving patient satisfaction; it must be combined with high-quality interpersonal care and service reliability. Policymakers must guarantee that healthcare innovations emphasise patient-centred care, concentrating on technology implementation and enhancing service quality and patient participation. Healthcare administrators must reconcile innovation with efficient patient-provider relationships by investing in staff training to improve technological and human-centred service delivery. Furthermore, healthcare practitioners should integrate patient feedback to synchronise service advancements with actual needs.

## Supplementary Information

Supplementary Material 1.

## Data Availability

The datasets used and/or analysed during the current study are available from the corresponding author on reasonable request.

## Abbreviations

PS: Patient satisfaction
SI: Service Innovation
SSA: sub-Saharan Africa
CO: Customer Orientation
SDGs: Sustainable Development Goals
IRB: Institutional Review Board
OUM: Open University Malaysia
AIT: Accra Institute of Technology
