# Supplementary material for: The mediating role of service innovation in the relationship between customer orientation and patient satisfaction
Source: BMC Health Serv Res. 2025 Jun 4;25:803. doi: 10.1186/s12913-025-12794-7 (PMC12139258; doi:10.1186/s12913-025-12794-7)
Supplement: Supplementary file 1 — Supplementary Material 1. [file 12913_2025_12794_MOESM1_ESM.docx]

### **APPENDIX**

### **QUESTIONNAIRE**

**PART I: BACKGROUND OF PATIENT** (Please Tick where appropriate)

1. Gender
   1. Male
   2. Female
2. Age Groups: a. 18-20

b. 21-30

c. 31-40

d. 41-50

e. 51-60

f. 61 and above

1. Education Level:
   1. Illiterate
   2. Primary School Graduate
   3. Secondary School Graduate
   4. High School Graduate
   5. University Graduate
   6. Master’s
   7. Others
2. Marital Status:
   1. Married
   2. Single
   3. Divorced
3. Occupation
4. Name of hospital

**PART II**

**SECTION A: CUSTOMER ORIENTATION**

*This section seeks to gauge the level of customer orientation in the hospital. On a scale of 1=Strongly Disagree to 5=Strongly Agree, please rate the extent to which you agree with each statement in this section.*

|  | **Customer Orientation** | **Strongly Disagree** | **Disagree** | **Neutral** | **Agree** | **Strongly Agree** |
| --- | --- | --- | --- | --- | --- | --- |
| 1 | I am excited about the range of services that the hospital provides |  |  |  |  |  |
| 2 | The hospital provides personalised services that meet my health preferences |  |  |  |  |  |
| 3 | Customer service is provided in an excellent manner by the hospital |  |  |  |  |  |
| 4 | The hospital works hard to satisfy patients |  |  |  |  |  |
| 5 | No matter how employees feel, they always do the best they can for every patient they serve |  |  |  |  |  |
| 6 | The hospital often makes an extra effort to help patients even if it is not expected of them |  |  |  |  |  |
| 7 | The hospital periodically finds out patient’s needs in order to serve them better |  |  |  |  |  |
| 8 | I am well-informed about the services being provided |  |  |  |  |  |
| 9 | I have high confidence in the services that the hospital provides. |  |  |  |  |  |
| 10 | The range of health services that the hospital provides is adequate. |  |  |  |  |  |

**SECTION B: SERVICE INNOVATION**

*This section seeks to gauge the level of service innovation in the hospital. On a scale of 1=Strongly Disagree to 5=Strongly Agree, please rate the extent to which you agree with each statement in this section.*

|  |  | **Strongly Disagree** | **Disagree** | **Neutral** | **Agree** | **Strongly Agree** |
| --- | --- | --- | --- | --- | --- | --- |
| 1 | The hospital provides modern treatment routines for patients |  |  |  |  |  |
| 2 | The hospital has a technology that simplifies the patient’s administration procedures. |  |  |  |  |  |
| 3 | The hospital has a modern technology that helps in diagnosing and treating  patients |  |  |  |  |  |
| 4 | Hospital staff ensures that the right tools and equipment are used in the provision of service. |  |  |  |  |  |
| 5 | The use of technology gives a priority in the provision of healthcare services at the hospital. |  |  |  |  |  |
| 6 | I have great confidence in the systems used by the hospital in the provision of services. |  |  |  |  |  |
| 7 | Services delivery at the hospital is fast and quick. |  |  |  |  |  |
| 8 | Poor systems are avoided by the hospital to ensure patient satisfaction. |  |  |  |  |  |
| 9 | The hospital uses innovation in the provision of services |  |  |  |  |  |
| 10 | I am satisfied with the innovative ways the hospital adopts in the provision of services. |  |  |  |  |  |

**SECTION C: PATIENT SATISFACTION**

*This section seeks to gauge the level of satisfaction with service delivery in the hospital. On a scale of 1=Strongly Disagree to 5=Strongly Agree, please rate the extent to which you agree with each statement in this section.*

|  |  | **Strongly Disagree** | **Disagree** | **Neutral** | **Agree** | **Strongly Agree** |
| --- | --- | --- | --- | --- | --- | --- |
| 1 | Overall I was satisfied with the physician’s level of availability in attending to me |  |  |  |  |  |
| 2 | Overall I was satisfied with the administrative procedures in the hospital |  |  |  |  |  |
| 3 | Overall I was satisfied with the treatment by the medical staff. |  |  |  |  |  |
| 4 | I was satisfied with the quality of services that the hospital provides. |  |  |  |  |  |
| 5 | I received the kind of services that I needed and preferred from the hospital. |  |  |  |  |  |

**THANK YOU.**
